# Selective Deposition of Mo_2_C-Containing Coatings on {100} Facets of Synthetic Diamond Crystals

**DOI:** 10.3390/ijms23158511

**Published:** 2022-07-31

**Authors:** Arina V. Ukhina, Boris B. Bokhonov, Dina V. Dudina

**Affiliations:** 1 Institute of Solid State Chemistry and Mechanochemistry, Siberian Branch of the Russian Academy of Sciences, Kutateladze Str. 18, 630090 Novosibirsk, Russia; bokhonov@solid.nsc.ru (B.B.B.); dina1807@gmail.com (D.V.D.); 2 Lavrentyev Institute of Hydrodynamics, Siberian Branch of the Russian Academy of Sciences, Lavrentyev Ave. 15, 630090 Novosibirsk, Russia

**Keywords:** diamond, carbide, molybdenum, coatings, selective deposition, scanning electron microscopy, X-ray diffraction, gas phase transport

## Abstract

An efficient way to improve the properties of metal–diamond composites (mechanical strength, wear resistance, thermal conductivity) is the preliminary modification of the diamond surface to improve its wettability by the metal matrix. In the present work, Mo_2_C-containing coatings were deposited on the diamond crystals under different conditions: hot pressing (atmosphere of argon), spark plasma sintering (forevacuum), and annealing in air. The influence of the sintering parameters on the morphology and phase composition of the coatings deposited on diamond was studied. Mo_2_C-containing coatings were selectively deposited on the facets of synthetic diamond microcrystals by annealing of the latter with a molybdenum powder. Experiments were carried out to deposit coatings under different conditions: during hot pressing (argon atmosphere), spark plasma sintering (forevacuum), and annealing in air. The process parameters were the temperature, holding time, and concentration of molybdenum in the initial mixture. Experiments with a pre-oxidized molybdenum powder were also conducted. The coated diamond crystals were investigated by X-ray diffraction, scanning electron microscopy, and energy-dispersive spectroscopy. The deposition was enabled by the gas phase transport of molybdenum dioxide, MoO_2_, contained in the starting powder. The following sequence of the coating formation stages was proposed. First, MoO_2_ sublimes and is adsorbed mainly on the {100} facets of diamond. Then, it is reduced to metallic molybdenum by carbon of the diamond, which further reacts with carbon to form the Mo_2_C carbide phase. These processes occurred during treatment of the mixtures in the hot press and the spark plasma sintering facility. When the mixture was annealed in air, no selective deposition was observed. During annealing, MoO_3_ particles adhered to the diamond surface.

## 1. Introduction

At present, novel diamond-based functional materials are being sought. Metal–diamond composites can be used as heat dissipating elements in high-power devices, in which the thermal contact area is small and the required power dissipation density is high. In these materials, copper is commonly used as a metallic component owing to its lower cost and higher thermal conductivity in comparison with other metals [1]. However, copper does not wet the diamond surface, which leads to the formation of pores at the interfaces and lowers the thermal conductivity of the composites. This problem can be solved by depositing coatings on the diamond surface. Several methods have been used to modify the diamond surface [2,3,4,5,6], which include magnetron sputtering and electroless plating [2], magnetron sputtering combined with annealing [3], and the vacuum micro-vapor deposition method [4]. The carbide-forming metals are mainly used for making the coatings [7,8,9].

One of the promising metals for coating diamond crystals to be further used in the copper–diamond composites with a high thermal conductivity is molybdenum [10]. In order to deposit molybdenum-containing coatings on the diamond surface, the molten salt method [10,11], magnetron sputtering [12], chemical vapor deposition [13], or vacuum micro-vapor deposition can be used [14]. Recently, a new method for obtaining coatings on powder particles (crystals) based on annealing of the powder mixtures (the substrate mixed with a coating precursor) in a spark plasma sintering (SPS) facility (coating of diamond by Ti) [15] or a hot press (HP) (coating of diamond by W) [16] has been proposed. This approach is characterized by a high efficiency and a relative simplicity of the technological process. An advantage of this process is the possibility of depositing coatings on powder particles or crystals without the formation of bulk compacts. Liquid phases are not involved in this process, which is another advantage, as their presence would lead to certain technological difficulties.

At present, the synthetic diamond crystals are abundant on the market. The most developed facets of the crystals are those with {100} and {111} indices. These facets differ in the packing pattern of the carbon atoms and surface energy [17,18]. Owing to the intrinsic differences between the {100} and {111} facets, the interactions of these facets with an external reactant may be expected to proceed differently. The effect of selective deposition of coatings on certain facets of the diamond crystals has been observed for silver [19], tungsten [16], and molybdenum [14]. As suggested in [16], oxides present on the surface of metallic particles can play a significant role in the process of the coating formation, if they are the volatile and enable the gas-phase transport of the coating precursor material to the diamond surface.

For the first time, Mo_2_C-containing coatings on the surface of diamond microcrystals were obtained by hot pressing in an atmosphere of argon, spark plasma sintering in forevacuum, and annealing in air using metallic molybdenum powder as a metal source. Factors influencing the phase and morphological features of the coatings were determined.

## 2. Results and Discussion

**Figure 1:**
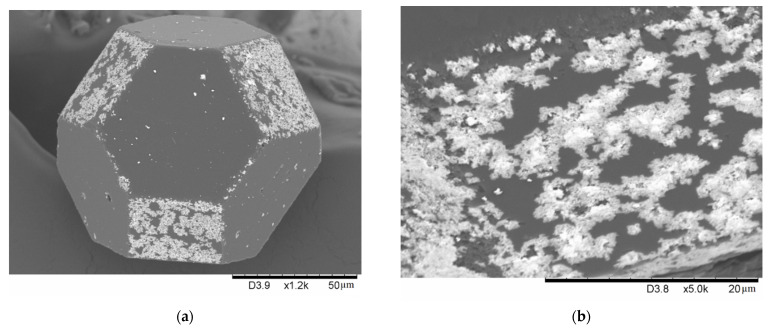

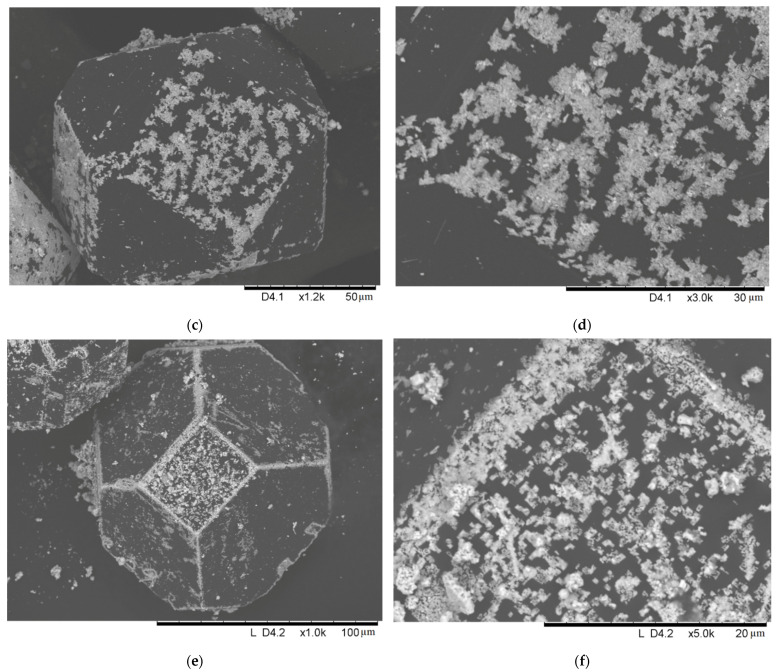
Surface of the diamond crystals: 10-900-5-HP (**a**), 10-900-15-HP (**c**), and 10-900-30-HP (**e**). Morphology of the Mo-containing island structures on the diamond surface on the {100} facets (**b**,**d**,**f**).

Figure 1 shows the morphology of the coatings obtained by treating the 10 vol.% Mo–diamond mixture in the HP at 900 °C for 5, 15, and 30 min. It can be seen that an island-like coating is formed predominantly on the {100} facets of diamond. As the holding time increases, the islands acquire a specific morphology.

**Figure 2:**
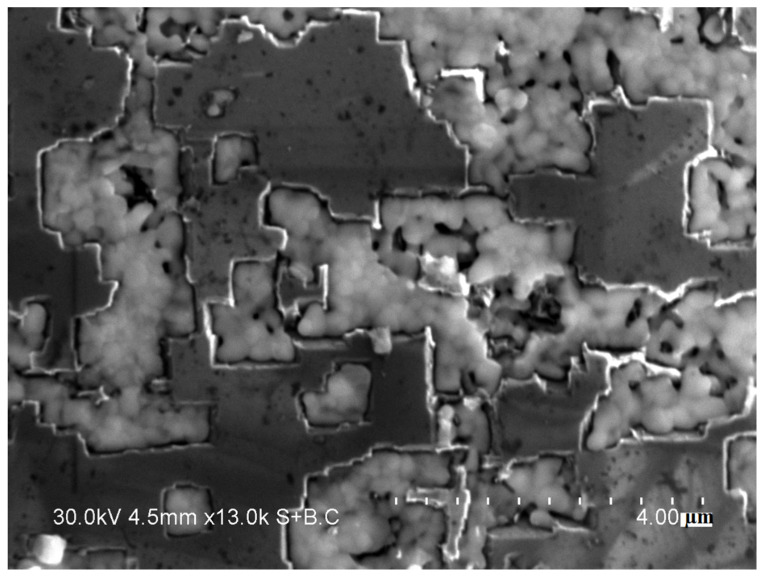
Morphology of the Mo-containing island structures on the {100} facets, 10-900-30-HP.

The coating islands obtained after 30 min of treatment in the HP have more distinct boundaries and are seen as squares and rectangles. The boundaries of these structures are almost parallel to the boundaries of the {100} facets (Figure 1f). Figure 2 shows a more detailed view of the morphology of these islands, confirming their polycrystalline nature.

**Figure 3:**
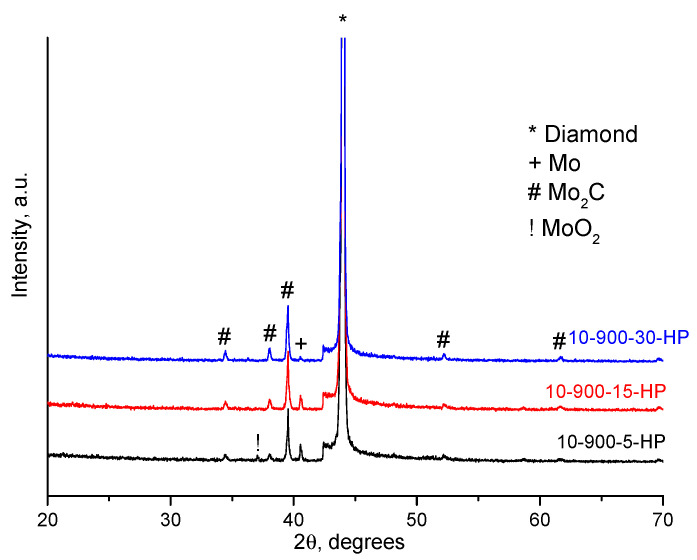
XRD patterns of the samples obtained at 900 °C (different holding times).

According to the XRD analysis (Figure 3), the coatings mainly consist of molybdenum carbide, Mo_2_C, and molybdenum, Mo. The concentration of the carbide increased and that of molybdenum decreased with increasing treatment time. After 5 min of treatment, the mixture demonstrates reflections of the MoO_2_ phase.

**Figure 4:**
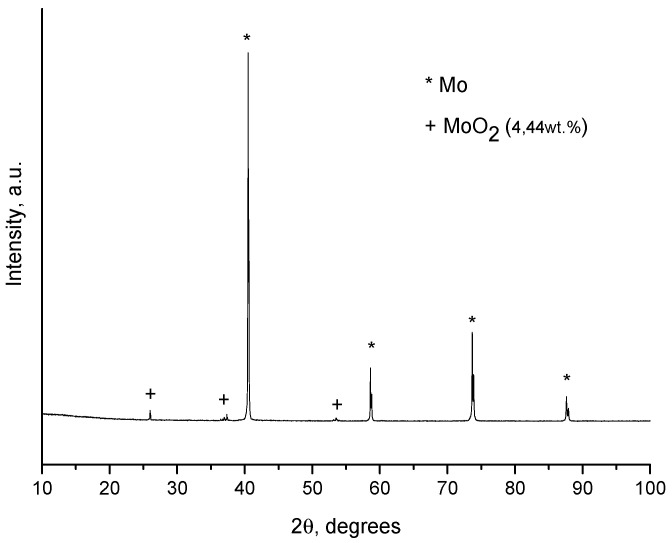
XRD pattern of the starting Mo powder.

Since the coatings were deposited on the certain facets of the diamond only, we can assume that they formed via the gas phase transport of Mo-containing species. The molybdenum dioxide, MoO_2_, which was present in the initial molybdenum powder, is volatile at the selected treatment temperature. Sublimation of the oxide was followed by its deposition on the certain facets of diamond. The concentration of MoO_2_ calculated from the XRD pattern of the starting molybdenum powder (Figure 4) was ~5 wt.%. The selective deposition of the oxide can be due to a difference between the surface energies of the diamond facets with different indices [20]. The coating formation process can be described by the following sequence of stages. First, volatile MoO_2_ sublimes and is deposited (adsorbed) on the diamond surface, mainly on the {100} facets. As the annealing time increases, MoO_2_ is reduced to metallic molybdenum by carbon of the diamond. At the same time, the Mo_2_C phase forms. After annealing for 30 min, MoO_2_ is almost completely consumed by the formation of Mo_2_C.

**Figure 5:**
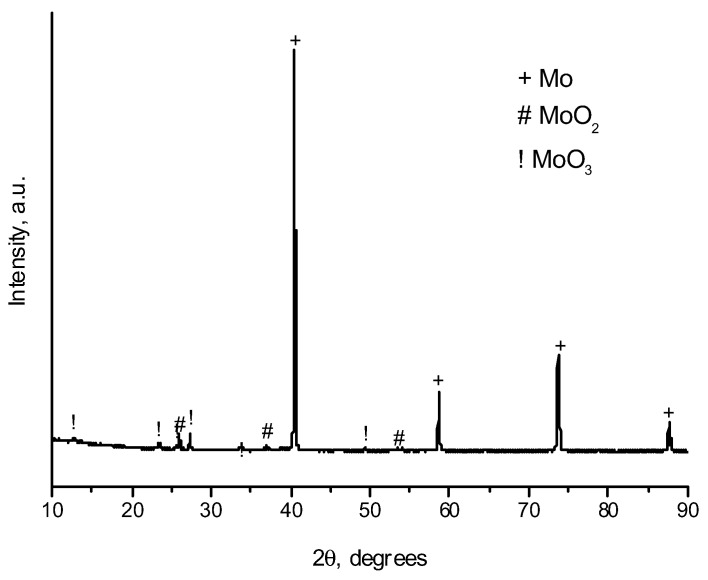
XRD pattern of the partially oxidized Mo powder.

**Figure 6:**
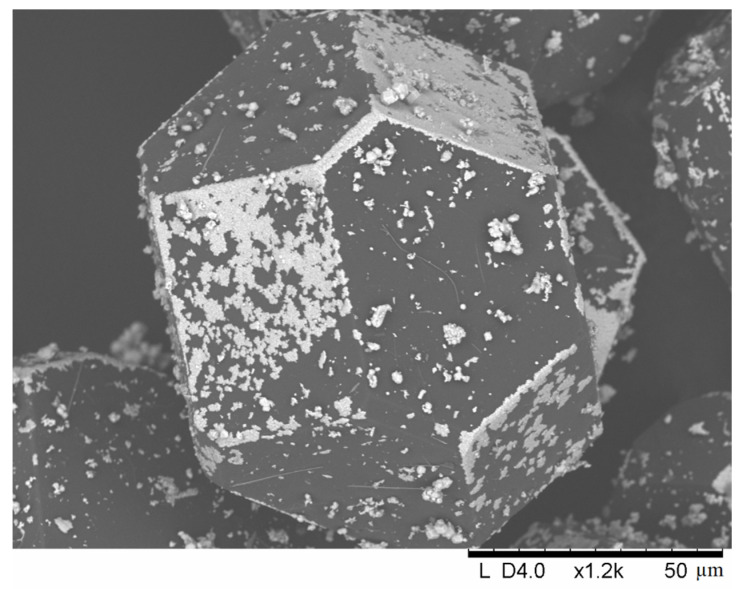
Surface of a diamond crystal, 10-900-30-ox-HP.

**Figure 7:**
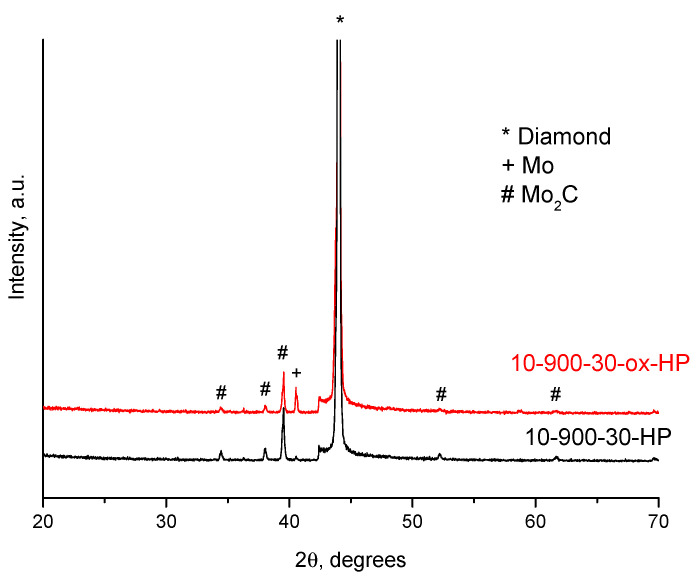
XRD patterns of the samples obtained at 900 °C, treatment for 30 min, with use of Mo and Mo(ox) as a metal source.

Assuming the coating formation is enabled by the gas-phase transport of the oxide, which is volatile at elevated temperatures, an additional experiment with a partially oxidized molybdenum powder was carried out. According to the XRD analysis, MoO_3_ formed upon oxidation (Figure 5). In the oxidized powder, the calculated content of Mo, MoO_2_, and MoO_3_ was 77 wt.%, 4 wt.%, and 19%, respectively. XRD peaks corresponding to MoO_3_ are broadened, which indicates a small crystallite size of the oxide. With the use of the pre-oxidized powder, the selectivity of the coating deposition on the diamond facets is retained (Figure 6). An increase in the concentration of the oxide in the powder mixture leads to an increase in the concentration of the oxides in the gas phase during the treatment. As seen in Figure 7, the phase composition of the coating obtained from the partially oxidized molybdenum powder was different from that obtained from the as-received powder. In the case of the pre-oxidized powder, the concentration of metallic molybdenum in the coating was significantly higher. This effect may be due to the fact that reduction of molybdenum oxides to metallic molybdenum is much faster than carbidization of metallic molybdenum. The carbidization reaction occurs slowly, governed by diffusion through the already-formed carbide layer on the diamond surface.

**Figure 8:**
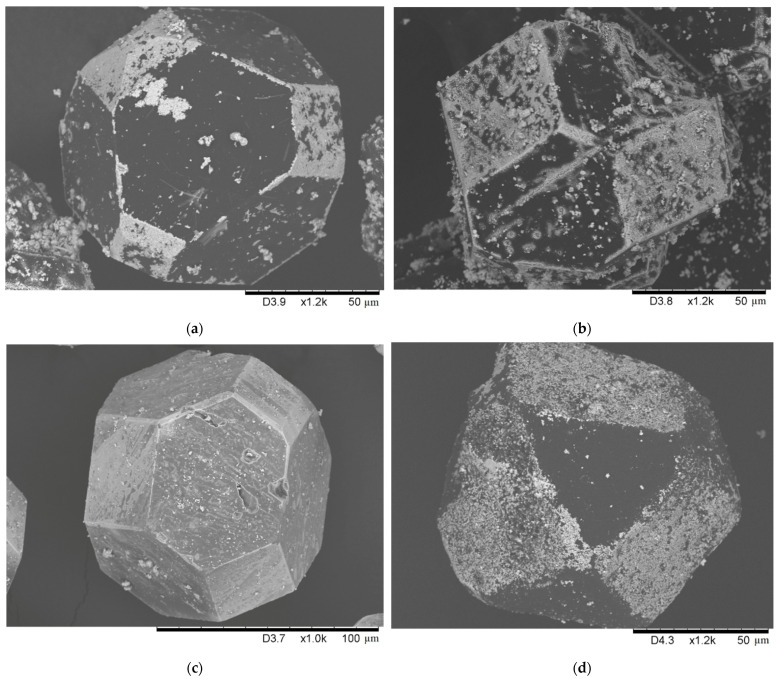
Surface of the diamond crystals: 10-1000-15-HP (**a**), 10-1000-30-HP (**b**),10-1000-15-SPS (**c**), and 50-1000-15-HP (**d**).

**Figure 9:**
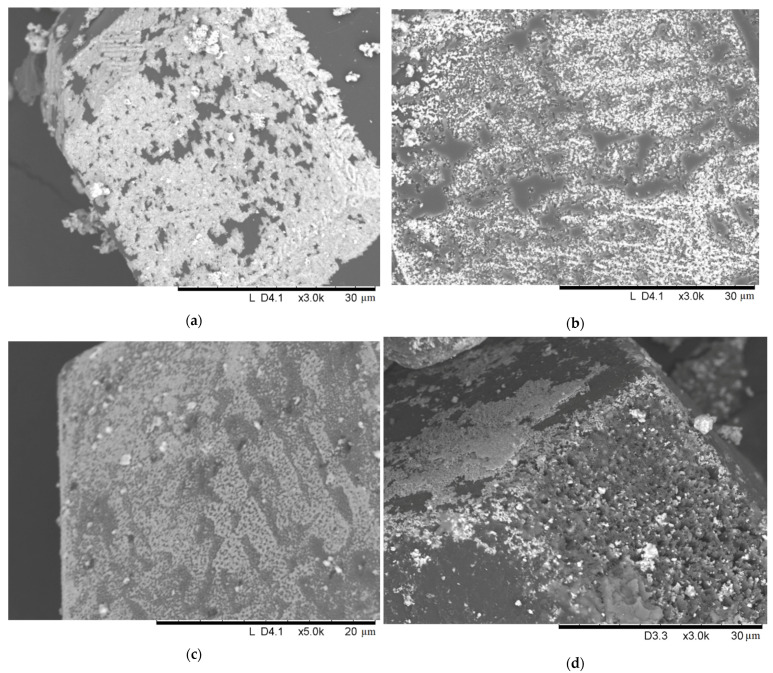
Surface morphology of a {100} diamond facet: 10-1000-15-HP (**a**), 10-1000-30-HP (**b**), 10-1000-15-SPS (**c**), and 50-1000-15-HP (**d**).

Another series of experiments were carried out at 1000 ℃. As seen in Figure 8a,b, the coating morphology on the diamond surface was similar to that obtained at 900 ℃. When the concentration of molybdenum in the starting mixture was increased to 50 vol.%, the selectivity of the coating was still retained: the {100} diamond facets were coated to a greater extent than the {111} facets (Figure 8d). At the same time, the coating microstructure changed: on the {100} facets, surface “etching” was observed, resulting in the formation of numerous etching pits (Figure 9d).

The Mo_2_C coating formation scheme can be presented as follows:

MoO_2_ (g) + C (s) → Mo (s) + CO_2_ (g)↑ (1)

2Mo (s) + C (s) → Mo_2_C (s) (2)

Interestingly, the morphology of the coating obtained in the SPS chamber differed significantly from that of the coating obtained in the HP unit under the same measured temperature (compare Figure 8a,c). The coating becomes more homogeneous and the deposition selectivity onto the certain diamond facets is practically absent. This difference can, in part, be explained by the different heating mechanisms during the SPS and HP treatments. Heating by a passing electric current during SPS leads to local overheating at the inter-particle contacts [21,22], which, in turn, accelerates chemical reaction. The atmosphere in which the treatment was carried out, forevacuum during SPS and argon at an atmospheric pressure during HP, can also influence the deposition process of the coating.

**Figure 10:**
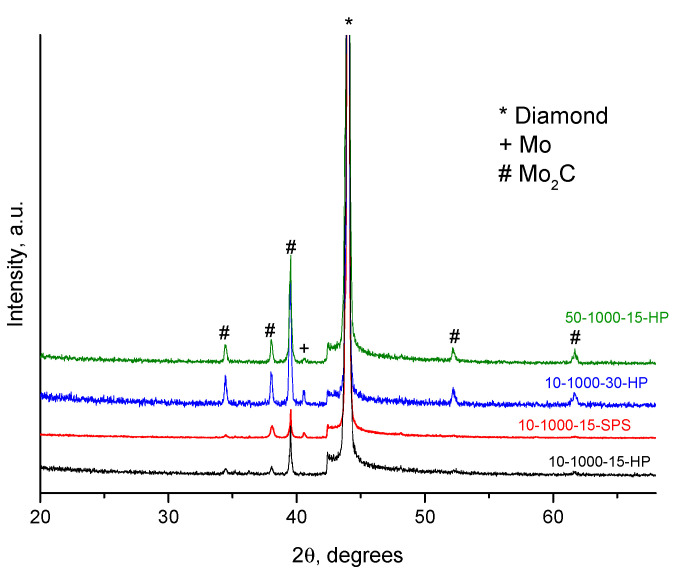
XRD patterns of the samples obtained at 1000 °C.

According to the XRD, all coatings obtained at this temperature consisted of molybdenum carbide (Mo_2_C) and molybdenum (Mo) (Figure 10). However, the carbide/metal ratio is different in these samples. The highest concentration of the carbide phase was found in 10-1000-30-HP, the sample subjected to a prolonged treatment in the HP. The holding time during the treatment is an important parameter for implementing the synthesis of the carbide by a slow carbidization reaction.

**Figure 11:**
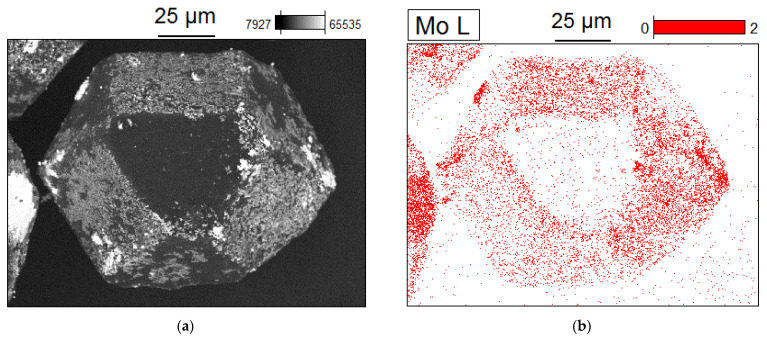
Scanning electron microscopy images (**a**) and elemental maps (**b**) of 10-1000-15-HP: a single coated diamond crystal, molybdenum-rich areas are red.

Figure 11 shows the element distribution on a coated crystal in 10-1000-15-HP. The data are in good agreement with the fact that molybdenum is deposited predominantly on the {100} facets of diamond.

**Figure 12:**
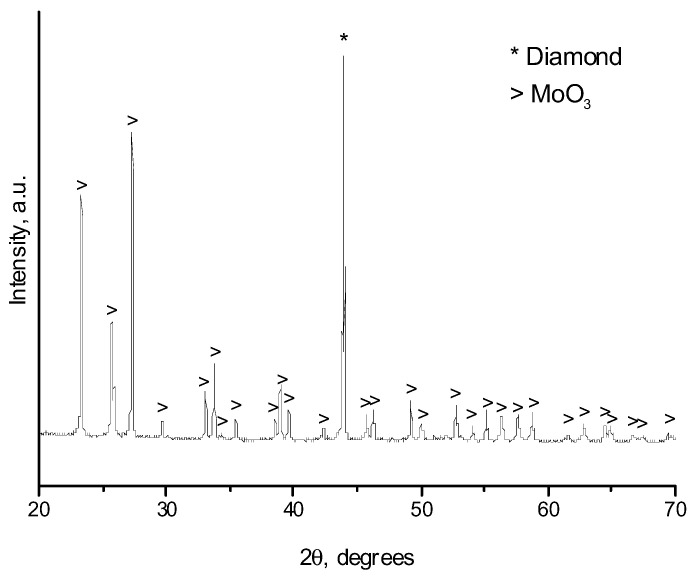
XRD pattern of the sample obtained at 650 °C, 60 min in air.

**Figure 13:**
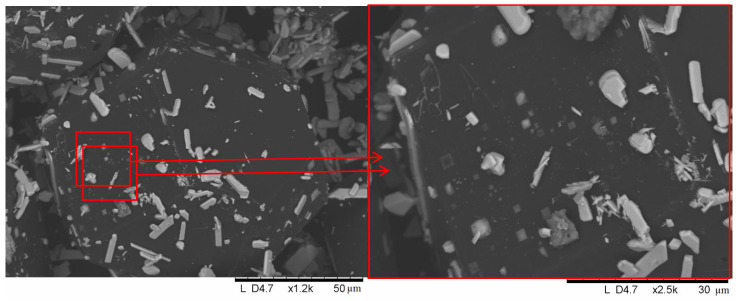
Surface of the diamond crystals with MoO_3_ particles, 50-650-60-air, different magnifications.

When a mixture of molybdenum powders and diamond is heated in air, both the phase composition of the coating and its morphology differ from those obtained by HP or SPS treatment. According to XRD, in this case, only molybdenum oxide (MoO_3_) was present on the diamond surface (Figure 12), which indicates complete oxidation of the molybdenum powder under the selected conditions. Figure 13 shows the images of the particles on the surface of the diamond recorded at different magnifications. The electron microscopy analysis shows that no selective deposition occurred during annealing. The particles were uniformly distributed over the facets of the diamond crystals, showing that deposition was not selective under those conditions. At a high magnification, in addition to the oxide particles, islands (squares and rectangles) with boundaries parallel to the edges of the {100} facets could be seen on the diamond surface.

## 3. Materials and Methods

Synthetic diamond D (MBD12, Changsha, China, average particle size 100 μm) and molybdenum Mo (MPCh, 99.1%, Moscow, Russia) powders were used as the starting materials. The coatings were deposited via treatment of the mixtures in HP and SPS facilities. A hot press (a custom-made facility developed by the Institute of Automation and Electrometry SB RAS, Novosibirsk, Russia) and a Labox 1575 apparatus (SINTER LAND Inc., Nagaoka, Japan) were used for conducting the treatment.

The powder mixtures containing 10 vol.% and 50 vol.% of Mo were prepared by thoroughly mixing the diamond and Mo powders in a mortar. Mixtures were placed in a graphite die with an inner diameter of 10 mm. The inner walls of the die and flat ends of the punches were protected by a layer of graphite foil. Mixtures with 10 vol.% of Mo were heated in the HP in an argon atmosphere (pressure of argon 20 kPa) up to 900 °C and held for 5, 15, and 30 min. They were also heated up to 1000 °C and held for 15 and 30 min. The same mixture was heated in the SPS chamber up to 1000 °C (in forevacuum, residual pressure 10 Pa) and held for 15 min at this temperature. The mixture with 50 vol.% of Mo was heated up to a temperature of 1000 °C in the HP, the holding time was 15 min. In addition, this mixture was heated in air up to 650 °C and held for 60 min at that temperature. The heating rate was constant in all experiments and was equal to 50 °C min^−1^. The uniaxial pressure applied to the samples was 10 MPa for all experiments.

In order to determine the effect of the concentration of the oxide phases in the starting mixture, experiments with a pre-oxidized molybdenum powder were carried out. For that, the molybdenum powder was first annealed in air at 400 °C for 30 min. Then, the partially oxidized powder was mixed with the diamond crystals. The weight percentage of the oxidized powder in the mixture was taken equal to that in the 10 vol.% Mo–diamond mixture. The mixture was treated in the HP at 900 °C for 30 min. After the treatments, the powders were sieved (through a sieve with a size of openings of 80 μm) to separate the diamond particles. The sample notations are presented in Table 1.

The morphology of the coatings on the diamond microcrystals was studied by scanning electron microscopy using a Hitachi TM-1000 Tabletop Microscope (Japan). Energy-dispersive spectroscopy (EDS) and elemental mapping was conducted using a NORAN Spectral System 7 unit (Thermo Fisher Scientific Inc., Waltham, MA, USA) attached to a S-3400N microscope (Hitachi, Chiyoda-ku, Japan). The phase composition of the samples was studied using X-ray diffraction (XRD). The XRD patterns of the samples were recorded on a D8 ADVANCE X-ray diffractometer (Bruker AXS, Karlsruhe, Germany). For the quantitative phase analysis, the Rietveld method was used (TOPAS 4.2 software, Bruker AXS, Karlsruhe, Germany).

## 4. Conclusions

Mo_2_C-containing coatings on the surface of synthetic diamond microcrystals were obtained by treatment of the mixtures of molybdenum and diamond powders in the HP and SPS facilities. The influence of the processing conditions (atmosphere, temperature, and holding time) on the phase composition and morphology of the coatings was studied. During treatment at 900 °C in the HP in an argon atmosphere, the coatings formed mainly on the {100} diamond facets. When the treatment temperature was increased up to 1000 °C, the deposition selectivity was retained. When the treatment time was increased, the selectivity was retained, but the phase composition of the coating changed: the concentration of Mo_2_C in the coating increased. A homogeneous coating was obtained by SPS in forevacuum. Based on results of the experiments, the process of the coating formation can be described by the following stages. First, MoO_2_ sublimes and is adsorbed mainly on the {100} facets of diamond. Then, it is reduced to metallic molybdenum by carbon of diamond, which further reacts with carbon to form Mo_2_C. When the mixture was annealed in air, the molybdenum powder was fully oxidized. The molybdenum oxide particles (MoO_3_) were uniformly distributed over the facets of the diamond crystals, showing that deposition was not selective under those conditions. The results of the present work allow deepening our understanding of the chemical processes occurring during the formation of metal and carbide coatings on diamond, which is necessary for the development of composite materials with a high thermal conductivity. The novelty of the obtained results is in determining the microstructural and morphological features of Mo_2_C-containing coatings forming on the diamond surface during HP and SPS treatments of diamond–molybdenum mixtures.

## Figures and Tables

**Table 1:** Synthesis conditions and sample notations.

Sample Notation	Mo Concentration in the Starting Mixture, Vol.%	Treatment Temperature, °C	Treatment Time, min	Treatment Method
10-900-5-HP	10	900	5	HP
10-900-15-HP	10	900	15	HP
10-900-30-HP	10	900	30	HP
10-1000-15-HP	10	1000	15	HP
10-1000-30-HP	10	1000	30	HP
50-1000-15-HP	50	1000	15	HP
10-1000-15-SPS	10	1000	15	SPS
10-900-30-ox-HP	10 *	900	30	HP
50-650-60-air	50	650	60	Annealing in air

* the weight percentage of the oxidized powder in the mixture was taken equal to that in the 10 vol.% Mo–diamond mixture.

## Data Availability

Not applicable.
